# Inhibition of IL17A Using an Affibody Molecule Attenuates Inflammation in ApoE-Deficient Mice

**DOI:** 10.3389/fcvm.2022.831039

**Published:** 2022-02-24

**Authors:** Ashok Kumar Kumawat, Mulugeta M. Zegeye, Geena Varghese Paramel, Roland Baumgartner, Anton Gisterå, Obed Amegavie, Sanna Hellberg, Hong Jin, April S. Caravaca, Leif Å. Söderström, Lindvi Gudmundsdotter, Fredrik Y. Frejd, Liza U. Ljungberg, Peder S. Olofsson, Daniel F. J. Ketelhuth, Allan Sirsjö

**Affiliations:** ^1^Cardiovascular Research Centre, School of Medical Sciences, Örebro University, Orebro, Sweden; ^2^Division of Cardiovascular Medicine, Department of Medicine, Solna, Centre for Molecular Medicine, Karolinska University Hospital, Karolinska Institutet, Stockholm, Sweden; ^3^Affibody AB, Solna, Sweden; ^4^Department of Cardiovascular and Renal Research, Institute of Molecular Medicine, University of Southern Denmark, Odense, Denmark

**Keywords:** CXCL1, Affibody molecule, atherosclerosis, apolipoprotein E-deficient (ApoE^−/−^) mice, human aortic smooth muscle cells

## Abstract

The balance between pro- and anti-inflammatory cytokines released by immune and non-immune cells plays a decisive role in the progression of atherosclerosis. Interleukin (IL)-17A has been shown to accelerate atherosclerosis. In this study, we investigated the effect on pro-inflammatory mediators and atherosclerosis development of an Affibody molecule that targets IL17A. Affibody molecule neutralizing IL17A, or sham were administered *in vitro* to human aortic smooth muscle cells (HAoSMCs) and murine NIH/3T3 fibroblasts and *in vivo* to atherosclerosis-prone, hyperlipidaemic ApoE^−/−^ mice. Levels of mediators of inflammation and development of atherosclerosis were compared between treatments. Exposure of human smooth muscle cells and murine NIH/3T3 fibroblasts *in vitro* to αIL-17A Affibody molecule markedly reduced IL6 and CXCL1 release in supernatants compared with sham exposure. Treatment of ApoE^−/−^ mice with αIL-17A Affibody molecule significantly reduced plasma protein levels of CXCL1, CCL2, CCL3, HGF, PDGFB, MAP2K6, QDPR, and splenocyte mRNA levels of *Ccxl1, Il6*, and *Ccl20* compared with sham exposure. There was no significant difference in atherosclerosis burden between the groups. In conclusion, administration of αIL17A Affibody molecule reduced levels of pro-inflammatory mediators and attenuated inflammation in ApoE^−/−^ mice.

## Introduction

Innate and adaptive immunity are key in the formation and progression of atherosclerotic lesions and in plaque rupture (1, 2). Lipid loaded macrophages and T cells accumulate in the vascular wall and promote initiation and progression of atherosclerosis (1).

Interleukin (IL)-17A plays a critical role in the development of various chronic inflammatory diseases including inflammatory bowel disease, rheumatoid arthritis, and experimental autoimmune encephalitis through the actions of CD4^+^ T helper 17 (Th17) cells (3). IL17A also controls the immune responses against extracellular bacteria and fungi (3). IL17A is mainly produced by CD4^+^ Th17 cells. IL17A is also produced by other cells including CD8^+^ T cells, Yδ T cells, natural killer T cells, natural killer cells and neutrophils (4, 5). Th17 cells and Yδ T cells are the main producers of IL17A in atherosclerosis in ApoE^−/−^ mice (6).

A higher frequency of dual IFNγ^+^/IL17A^+^ producing T cells has been observed in human atherosclerotic coronary arteries (7). Increasing numbers of Th17 cells have been found in atherosclerotic plaques in experimental atherosclerosis (8) and patients with acute myocardial infarction and unstable angina (9). It has been proposed that IL17A contributes to atherogenesis by inducing vascular and systemic inflammatory responses, and inhibition of IL17A with αIL17A antibodies ameliorates experimental atherosclerosis (6, 8, 10).

Recently an Affibody molecule against IL17A (ABY-035) was developed and formatted to have very high potency and long half-life, that have been shown to reduce inflammation in patients with psoriasis (unpublished data). Affinity proteins are promising tools in the development of next generation therapeutics. Currently there are various alternative ways of blocking target molecules that are under development, including non-antibody derived small protein molecules called “Affibody molecules” (11, 12). Due to their small size compared to antibodies (6.5 vs. 150 kDa), these molecules show excellent tissue penetration, can be administered at high molar concentrations in a small volume for high dose subcutaneous administration, and have high affinity and specificity. Affibody molecules are not derived from antibodies and do not contain the fragment crystallizable (Fc) portion, which reduces unwanted side-effects such as antibody-dependent cell cytotoxicity (ADCC) and complement activation (11).

In the present study, we investigated the capacity of an isotype surrogate Affibody molecule cross-reactive with human and murine IL17A to reduce levels of inflammatory cytokines, plasma lipids, and atherosclerotic lesion development.

## Materials and Methods

### Cell Culture and *in vitro* Experiments

Primary human aortic smooth cells (SMCs, Life technologies, USA), were cultured in 75 cm^2^ flasks (Sarstedt, Germany) containing a basal vascular smooth muscle cell growth medium 231 (Gibco, Life Technologies, USA). The culture medium was supplemented with Smooth Muscle Growth Supplement (SMGS), and antibiotics [Penicillin (0.1 U/ml) + Streptomycin (100 ng/ml)-PEST, Gibco, Life Technologies]. Mouse fibroblast cells (NIH/3T3 cells) were maintained in DMEM medium (Lonza Ltd, Switzerland) supplemented with 10% FBS+ PEST (Gibco, Life Technologies). The cell cultures were maintained at 37°C in humidified air containing 5% CO2, by replacing medium every 2 days and/or sub-culturing upon confluence.

For setting up stimulation experiments human aortic SMCs (3 x 10^4^) and mouse NIH/3T3 cells (5 x 10^4^) were seeded and cultured overnight in respective complete media. The next day, the media was replaced with fresh antibiotics free medium, and cells were treated with different concentrations of human or murine recombinant IL17A (R&D systems, USA) and cultured in the presence or absence of human or murine Affibody molecule at different concentrations to block IL17A (Affibody AB, Sweden), for 24 h. Unstimulated cells served as reference. At the end of incubations, the supernatants were collected and stored at −80°C until further analysis.

### Animals

Male ApoE^−/−^ mice 10 weeks of age on C57BL/6J background were maintained within the Animal Research Facility at Karolinska Institute, Stockholm. Mice were fed a normal chow diet. A pharmacokinetics-pharmacodynamics (PK/PD) simulation approach was used to determine the dosage regimen for the Affibody molecule. A simulation of the exposure levels of Affibody molecule was performed using a one-compartment model in the PK/PD simulation software Maxsim2 (Fraunhofer–Chalmers Research Centre Industrial Mathematics). PK-parameters for the Affibody molecule were adopted from Affibody in-house data and the dose level was iterated in order to achieve sufficient exposure. The dosing regimen of 300μg/dose of the Affibody molecule, 2 times/ week for 10 weeks was estimated to yield an efficient trough- concentration of approximately 20 nM. Blocking anti-mouse albumin associating IL17A Affibody molecule (300 μg; Affibody AB, Stockholm, Sweden) or phosphate-buffered saline (PBS) as sham were administrated interperitoneally twice a week for 10 weeks, frequency and dose calculated to have stable and high steady state exposure given the Affibody molecule half-life of 24 h in mice (estimated to be approximately 12 days in man). All experiments were approved by the regional Ethical Committee on Animal Experiments Stockholm, Sweden.

### Tissue Processing, Immunohistochemistry, and Lesion Analysis

Mice were euthanized 1 week after the last injection of the αIL17A Affibody molecule or sham treatment. Blood samples were collected by cardiac puncture for plasma preparation and blood cell count and thereafter vascular perfusion was performed with sterile RNase-free PBS. The thoracic aorta was dissected and snap-frozen for later RNA isolation. The aortic arch was dissected and preserved for atherosclerotic lesion analysis in a 4% phosphate-buffered formaldehyde solution. The heart including the aortic root was removed and preserved by snap freezing for immunohistochemistry and quantitative lesion analysis. Spleens were collected and stored in PBS on ice until splenocyte isolation. Spleens were chopped on nylon mesh and passed through a 70 and 40 μm pore size nylon mesh strainer to a single cell splenocyte suspension. Isolated splenocytes were used for immunophenotyping of leukocytes by flowcytometry (BD FACS Verse flow cytometer (Becton Dickinson, NJ, USA). 8-10 × 10^6^ splenocytes were preserved in RNA later (Thermo Fischer Scientific, USA) and stored at −80°C until further analysis.

#### En Face Analysis of the Aortic Arch and Brachiocephalic Artery

Lipid accumulation in the mouse aortic arch and the brachiocephalic artery was determined as previously described (13). In brief, the aortic arch and its branches were cut open and pinned to a flat surface. Samples were stained using Sudan IV and micrographs of the aortas were captured using a microscope (Leica MZ6) and camera (Leica DC480) and stained area measured by a blinded evaluator. The stained plaque area was subsequently measured by a second blinded evaluator using ImageJ software (NIH). Plaque coverage was calculated as the percentage of plaque area of the total surface area of the aortic arch with the aortic intercostal arteries denoting a caudal end of the arch and not including branching vessels. Plaque coverage was measured similarly in the brachiocephalic artery.

#### Lesion Analysis in the Aortic Root

Quantification of atherosclerosis in the aortic root was performed as previously described (13). During the transport of frozen heart samples, tissues from 15 mice were damaged and we were thus able to perform aortic root staining in 8 mice only. In brief, the aortic root was sequentially sectioned at 10 μm thickness using a cryostat. Consecutive sections were cut in 100 μm intervals starting from the point at which all the three aortic valve leaflets appeared in the microscopic view. The aortic root sections were stained with Oil Red O followed by image acquisition using a Panoramic 250 Flash II (3DHISTECH Ltd, Hungary) scanner. Image J2 (NIH) software was used to quantify atherosclerotic lesion area, depending on quality of staining on slides, an average lesion area from 6 to 10 sections per mouse was calculated.

#### Determination of Blood Cell Count and Biochemical Profile in Plasma

Whole blood (EDTA) was analyzed on a Scil veterinary animal blood counter (Scil animal care company, Germany). Plasma was isolated by centrifugation at 1,500 g for 15 min. Total plasma cholesterol, high-density lipoprotein (HDL), low-density lipoprotein (LDL) and triglycerides were analyzed with a standard method by the Clinical Chemistry Laboratory, University Hospital Örebro. The concentration of glucose in plasma was determined using Mouse Glucose Assay Kit (Crystal Chem, USA) according to manufacturer's instruction.

### Flow Cytometry

Isolated splenocytes were exposed to anti-CD-16/32 (Biolegend, UK) for 5 min, then stained with antibodies CD45 AF647 (clone-30-F11), CD4 PE (clone-RM4-5), 7AAD (Biolegend), CD3 pacific blue (clone-500A2), CD8a V500 (clone-53-67) (BD Biosciences). The antibody staining was compared with fluorescence minus one (FMO) stain as negative control. Stained cells were analyzed on a BD FACS Verse flow cytometer (Becton Dickinson, NJ, USA). Data were analyzed using Kaluza software (Beckman Coulter).

### RNA Extraction, cDNA Synthesis, and Quantitative Real-Time PCR Analysis

Total RNA was extracted from the thoracic aorta and splenocytes. mRNA levels were measured by qPCR with PowerUp TM SYBR TM Green Master Mix (Thermo Fischer Scientific, USA) and with primers detailed in Supplementary Table 1 (Eurofins Genomics, Denmark). All samples were analyzed in duplicate, and the expression of each gene was normalized to the mRNA levels of beta-actin (β-actin). The relative expression values were calculated by the change-in-cycling-threshold method as 2^−*dΔC*(*t*)^.

### Olink Protein Analysis

Plasma samples were analyzed using olink protein analysis. The Mouse Exploratory panel (Olink Bioscience AB, Uppsala, Sweden) enables the analysis of 92 murine proteins related to a broad range of biological functions and pathways. The protein values are reported as linearized normalized protein expression levels (NPX). Proteins with signals below the limit of detection (LOD) were excluded from further analysis.

### ELISA

The release of IL6 and CXCL1(GRO-α) in cell culture supernatants were assayed by sandwich ELISA (R&D systems, USA) according to the manufacturer's protocol.

### Statistical Analysis

GraphPad Prism 9 was used to analyse the data (GraphPad Software Inc. USA). Based on distribution of data, comparisons between two groups were made by using Student's *t*-test or Mann-Whitney *U* two-tailed test as appropriate. Values are shown as mean ± SD or median with interquartile range (IQR). Correlation between the plasma concentration of proteins and the area of aortic root lesions was analyzed using Spearman correlation. *P* < 0.05 was considered significant. In cell culture experiments *n* = the number of independent experiments.

## Results

### αIL17A Affibody Molecule Blocks IL17A Induced CXCL1 and IL6 Release *in vitro*

**Figure 1:**
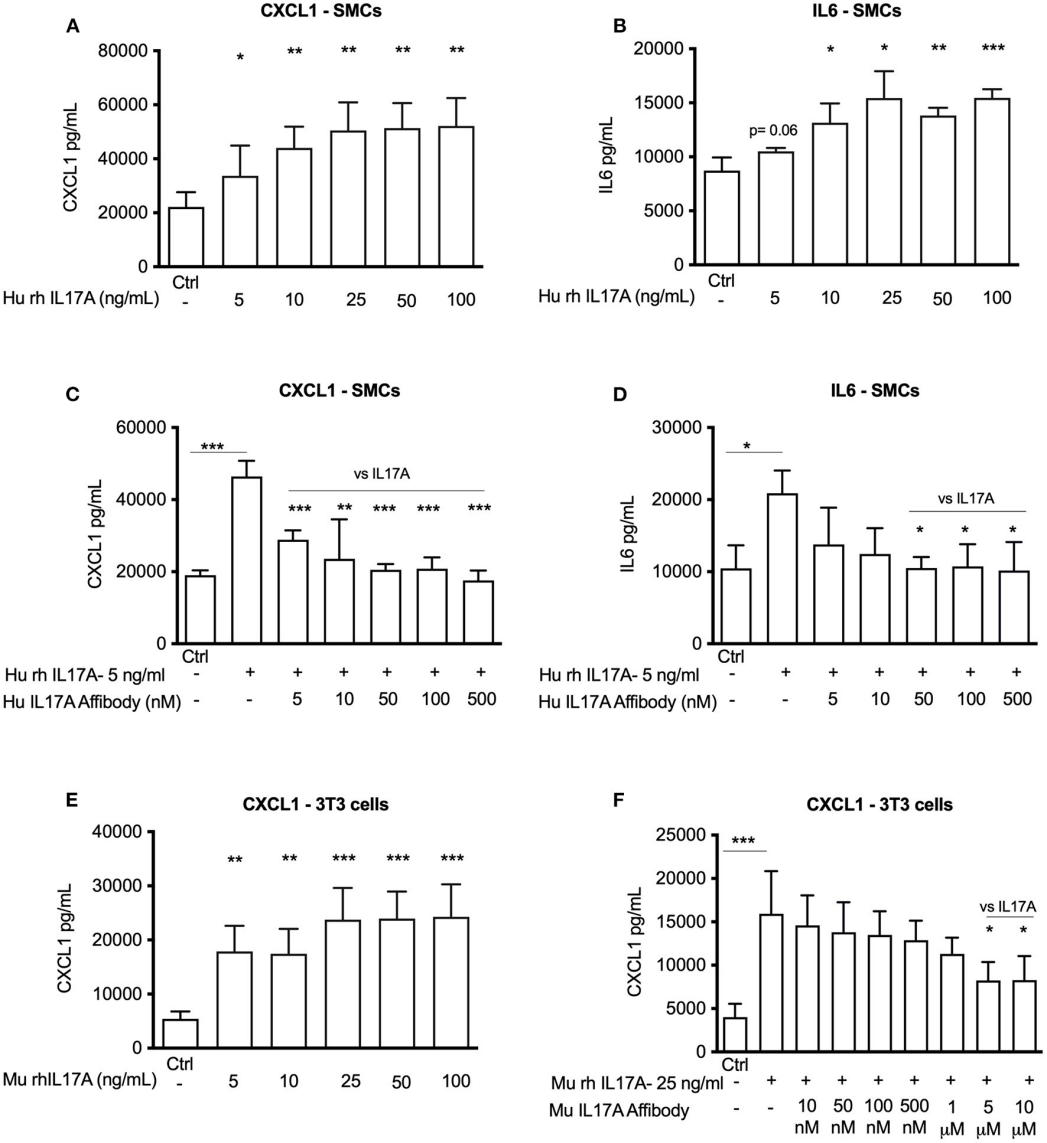
*In vitro* efficacy of Affibody molecules in blocking IL17A induced responses in human aortic smooth muscle cells (SMCs) and mouse NIH/3T3 fibroblast cells. ELISA data showing the release of CXCL1 and IL6 into the medium (24 h) induced by increasing concentration of recombinant IL17A in human SMCs (*n* = 4) **(A,B)** and CXCL1 in mouse 3T3 fibroblast cells (*n* = 4) **(E)**. Human SMCs were stimulated with 5 ng/ml recombinant IL17A and without or with increasing concentrations of human Affibody molecules against IL17A and release of CXCL1 and IL6 (24 h) was assessed by ELISA (*n* = 3) **(C,D)**. Mouse NIH/3T3 fibroblast cells were stimulated with 25 ng/ml recombinant IL17A and without or with increasing concentration of Affibody molecule against mouse IL17A and release of CXCL1 (24 h) was assessed by ELISA (*n* = 5) **(F)**. Data is presented as mean ± SD. *p < 0.05, **p < 0.01, ***p < 0.001 vs. control or as mentioned in graph (vs. IL17A).

IL17A is known to promote release of pro-inflammatory mediators CXCL1 and IL6 (7, 14, 15). Accordingly, we investigated whether IL17A promotes their release from human aortic SMCs. We observed that exposure of human aortic SMCs with 5 ng/ml of recombinant human IL17A significantly promoted release of CXCL1 and IL6 to the culture medium (Figures 1A,B). Administration of αIL17A Affibody molecule together with recombinant human IL17A significantly reduced release of both CXCL1 and IL6 in the culture medium (Figures 1C,D).

Similarly, recombinant murine IL17A (25 ng/ml) also significantly induced release of CXCL1 from murine NIH/3T3 cells (Figure 1E), while αIL17A Affibody molecule significantly reduced CXCL1 release from NIH/3T3 cells (Figure 1F). Together, these observations indicate that αIL17A Affibody molecule reduces IL17A-induced release of CXCL1 and IL6 in human SMCs and CXCL1-release in NIH/3T3 cells *in vitro*.

### Effect of αIL17A Affibody Molecule on Atherosclerotic Lesion Development in ApoE^–/-^ Mice

**Figure 2:**
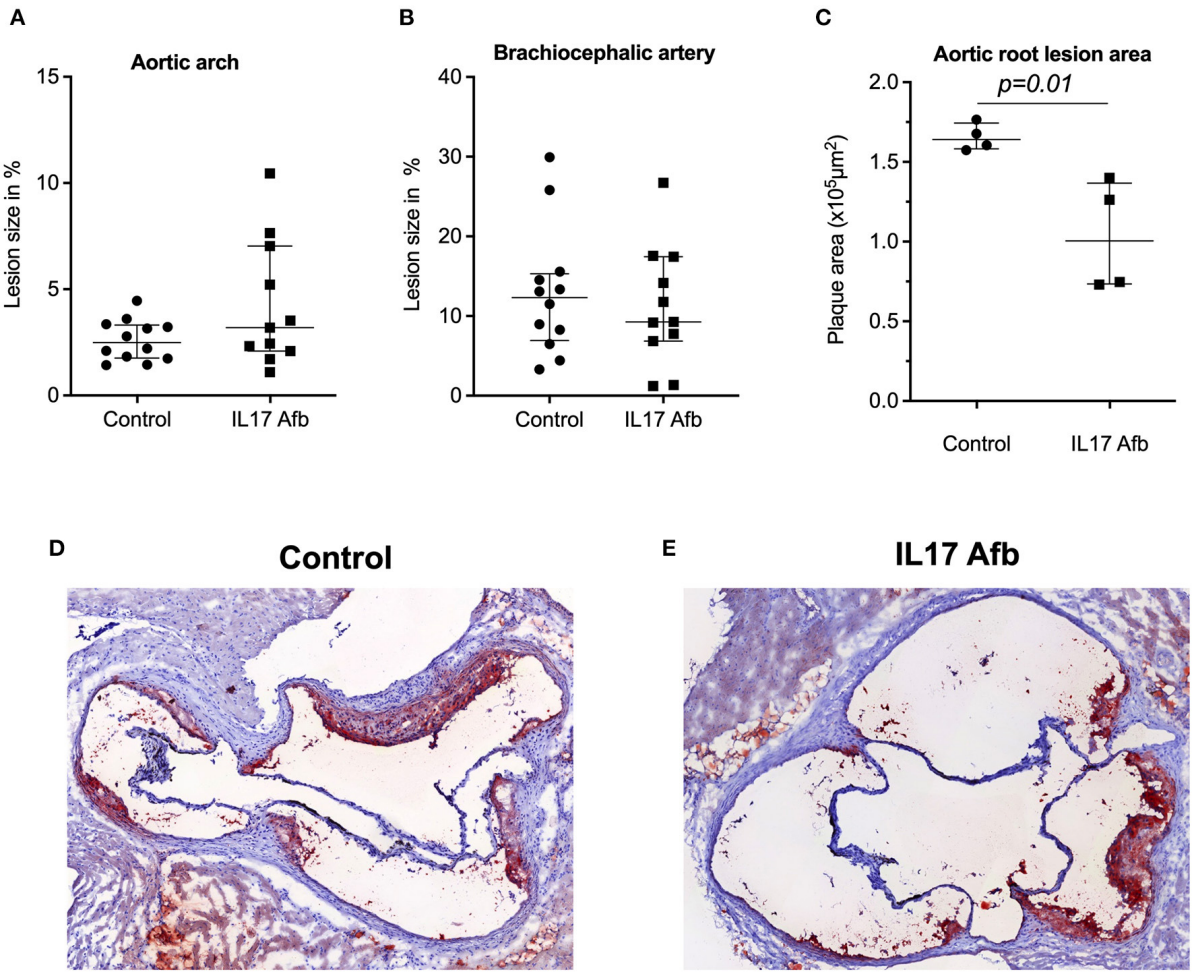
Effect of Affibody molecule targeting IL17A in atherosclerotic lesion development in ApoE^−/−^ mice. Quantification of lesion size (%) in the aortic arch **(A)** and brachiocephalic artery **(B)** of control mice and αIL17A Affibody molecule treated mice. Quatification of lesion areas (μm^2^) in the aortic root **(C)**. Representative Oil Red O immunostainings from the aortic root of control **(D)** and mice treated with αIL17A Affibody molecule **(E)**. Data are presented as Median with IQR.

To determine the effect of αIL17A Affibody molecule in development of atherosclerosis *in vivo*, we administered αIL17A Affibody molecule or PBS as sham interperitoneally in ApoE^−/−^ mice. There were no significant differences in the atherosclerotic lesion size as measured by *en face* staining in the aortic arch and brachiocephalic artery between αIL17A Affibody molecule and sham treated mice (*n* = 12) (Figures 2A,B). The atherosclerotic lesion size as measured by serial sectioning of the aortic root was significantly lower in αIL17A Affibody molecule compared with sham treated mice (*n* = 4) (Figures 2C–E).

### Treatment With αIL17A Affibody Molecule Did Not Significantly Change Blood Lipoprotein Levels or Spleen Cellular Composition in ApoE^–/-^ Mice

No animal died during the experimental period and mice that were treated with αIL17A Affibody molecule showed no significant growth retardation. Body weight, whole blood counts of leukocytes, plasma concentrations of cholesterol, HDL, LDL, and triglycerides were not significantly different between sham treated mice and mice injected with αIL17A Affibody molecule (Table 1). In addition, no significant changes were noted in plasma glucose levels between both groups (data not shown).

**Table 1:** Blood and morphometric parameters^a^ and T cell subsets in spleen^b^.

**Parameter**	**Control (mean ±SD)**	**IL17A Afb (mean ±SD)**	**p-value**
	**(*n* = 12)**	**(*n* = 10-11)**	
Leukocytes (10^9^/L)	7.46 ± 3.26	7.48 ± 1.98	n.s.
Monocytes (10^9^/L)	0.37 ± 0.22	0.37 ± 0.06	n.s.
Granulocytes (10^9^/L)	2.91 ± 2.14	2.46 ± 0.80	n.s.
Erythrocytes (10^12^/L)	3.64 ± 0.64	3.54 ± 0.37	n.s.
Hemoglobin (mmol/L	8.77 ± 1.36	8.90 ± 0.53	n.s.
Body weight (g)	33.25 ± 2.60	32.64 ± 2.30	n.s.
Triglycerides (mmol/L)	1.7 ± 0.54	1.58 ± 0.43	n.s.
Total cholesterol (mmol/L)	12.93 ± 2.54	11.42 ± 4.37	n.s.
LDL cholesterol (mmol/L)	1.57 ± 0.41	1.4 ± 0.64	n.s.
HDL cholesterol (mmol/L)	0.48 ± 0.16	0.64 ± 0.21	n.s.
**T cell composition in spleen** ^b^			
CD3^+^ (% of CD45^+^ cells)	25 ± 3	29 ± 4	n.s.
CD4^+^ (% of CD3^+^ cells)	52 ± 4	56 ± 4	p = 0.08
CD8^+^ (% of CD3^+^ cells)	2.91 ± 2.14	2.46 ± 0.80	n.s.
CD45^+^ total count (10^7^)	1.04 ± 0.36	1.04 ± 0.31	n.s.
CD3^+^ total count (10^6^)	2.59 ± 1.12	2.82 ± 1.06	n.s.
CD4^+^ total count (10^6^)	1.33 ± 0.58	1.54 ± 0.57	n.s.
CD8^+^ total count (10^5^)	6.71 ± 3.61	7.62 ± 3.97	n.s.

a *Body weight, hematological parameters, and lipid profile were measured on the day of tissue harvesting (20 weeks old) from mice treated with Affibody molecules against IL17A and sham*.

b *Proportion and total cell count of T cells (CD3^+^, CD4^+^, and CD8^+^) and total leukocyte count (CD45+) in splenocytes isolated from experimental animals and analyzed by using flow cytometry. All the values are presented as mean ± SD. n.s., not significant*.

Splenocytes were immunophenotyped using flow cytometry. The mean proportion and number of CD4^+^ T cells was increased in αIL17A Affibody molecule treated mice compared with sham treated mice, but the difference was not statistically significant (Table 1). The proportions and numbers of T cells (CD3^+^ and CD8^+^) and CD45^+^ leukocytes were not significantly different between groups (Table 1).

### αIL17A Affibody Molecule Reduced Plasma Protein Levels of Selected Inflammatory Markers in ApoE^–/-^ Mice

**Figure 3:**
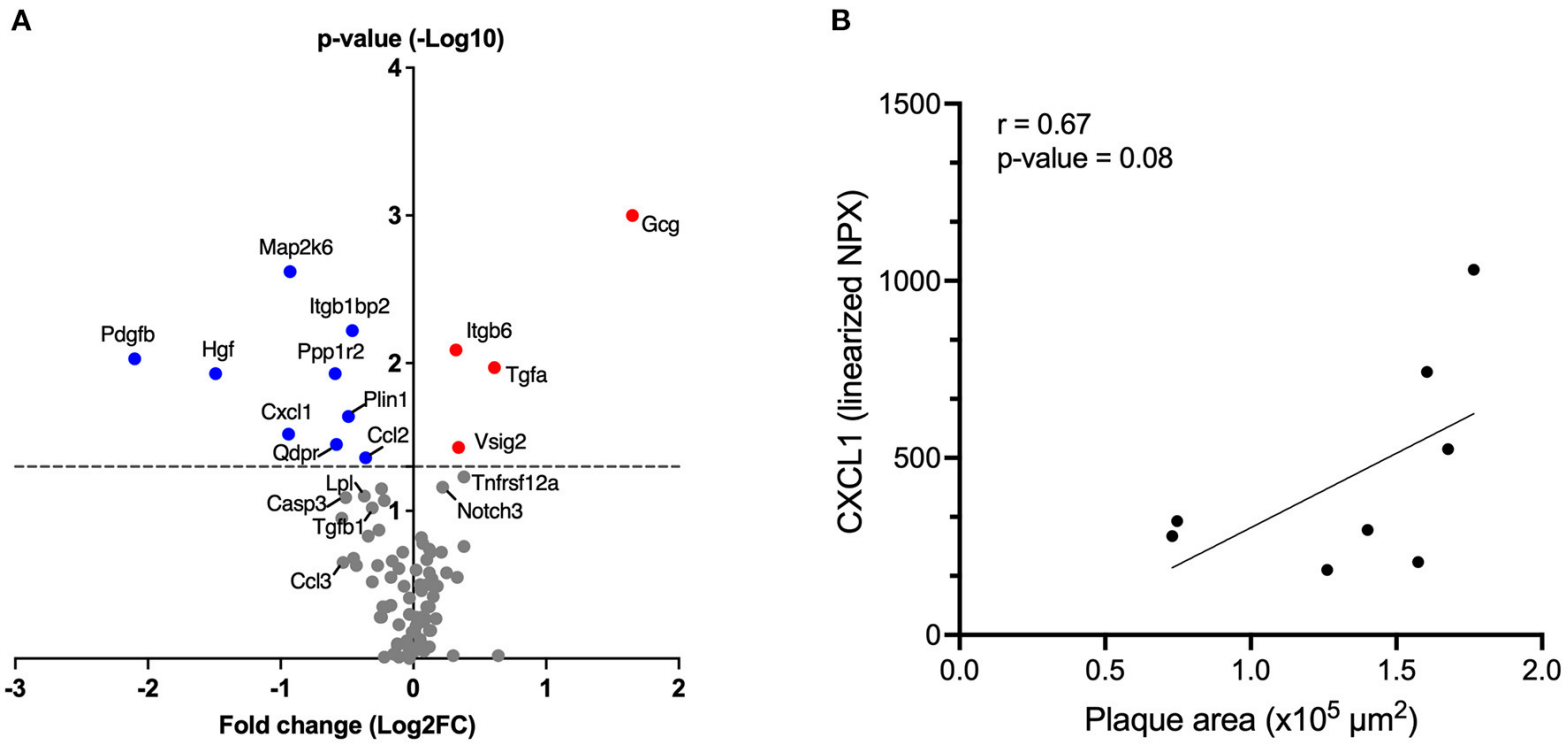
Affibody molecule against IL17A reduces plasma levels of inflammatory and/or atherosclerosis-associated proteins in ApoE^−/−^ mice. The Volcano plot shows fold changes (FC) and *p*-values of differentially altered plasma proteins analyzed by olink proteomics, in mice treated with αIL17A Affibody molecule (*n* = 11) compared to sham (*n* = 12) **(A)**. The *p*-values are presented on log10 scale while the FCs were calculated from linearized normalized protein expression (NPX) values and presented on log2 scale. Positive log2 fold change values correspond to higher protein levels and negative values correspond to reduced protein levels in mice treated with αIL17A Affibody molecule. The horizontal dotted line reflects the cut-off for statistical significance (*p* < 0.05). Blue circles highlight proteins with a significant decrease (*p* < 0.05) and red circles highlight proteins with a significant increase (*p* < 0.05). Gray circles represent proteins with no statistical significance. Correlation between the plasma concentration of CXCL1 protein and the area of aortic root lesion in atherosclerotic prone ApoE^−/−^ mice (*n* = 8) **(B)**.

Targeted protein analysis using olink proteomics showed that treatment with αIL17A Affibody molecule significantly reduced plasma protein levels of chemokines CXCL1 and CCL2 (MCP-1) compared with sham (Figure 3A and Supplementary Figure 1). Reduced CCL3 levels was also noted in the αIL17A Affibody molecule treated group compared with sham, but this difference was not statistically significant (Figure 3A and Supplementary Figure 1). Plasma levels of IL17A, IL6, CCL5, CCL20, CXCL9, and IL1-β were not significantly different between treatments (Supplementary Figure 2). In addition, there was a positive correlation between CXCL1 and lesion area in the aortic root (R^2^ = 0.67, *p* = 0.08), but this finding was not statistically significant (Figure 3B).

Furthermore, plasma levels of a set of proteins that others have associated with atherosclerosis development and other cardiovascular pathophysiology (e.g., growth factors and enzymes) (16–20) such as hepatocyte growth factor (HGF), platelet-derived growth factor B (PDGFB), MAP kinase 6 (MAP2K6), quinonoid dihydropyridine reductase (QDPR), protein phosphatase 1 regulatory inhibitor subunit 2 (PPP1R2), perilipin 1 (PLIN1) and integrin subunit beta 1 binding protein 2 (ITGBP1BP2) were significantly reduced in the αIL17A Affibody molecule treated group compared with sham (Figure 3A and Supplementary Figure 1).

We also noted reduced caspase 3 (CASP3), transforming growth factor (TGF)-β, lipoprotein lipase (LpL) protein levels in αIL17A Affibody molecule treated mice compared with sham, but this finding was not statistically significant (Figure 3A and Supplementary Figure 1).

In addition, significantly higher levels in plasma of glucagon (GCG), integrin β6 (ITGB6), TGFα, V-Set and immunoglobulin domain containing 2 (VSIG2), were also observed in αIL17A Affibody molecules treated mice compared with sham (Figure 3 and Supplementary Figure 3). Protein levels of NOTCH3 and TNF receptor super family member 12A (TNFRSF12A) were also increased in αIL17A Affibody molecule treated mice compared with sham, but this difference was not statistically significant (Figure 3 and Supplementary Figure 3).

### αIL17A Affibody Molecule Reduced mRNA Levels of Cxcl1 and Il6 in Splenocytes of ApoE^–/-^ Mice

**Figure 4:**
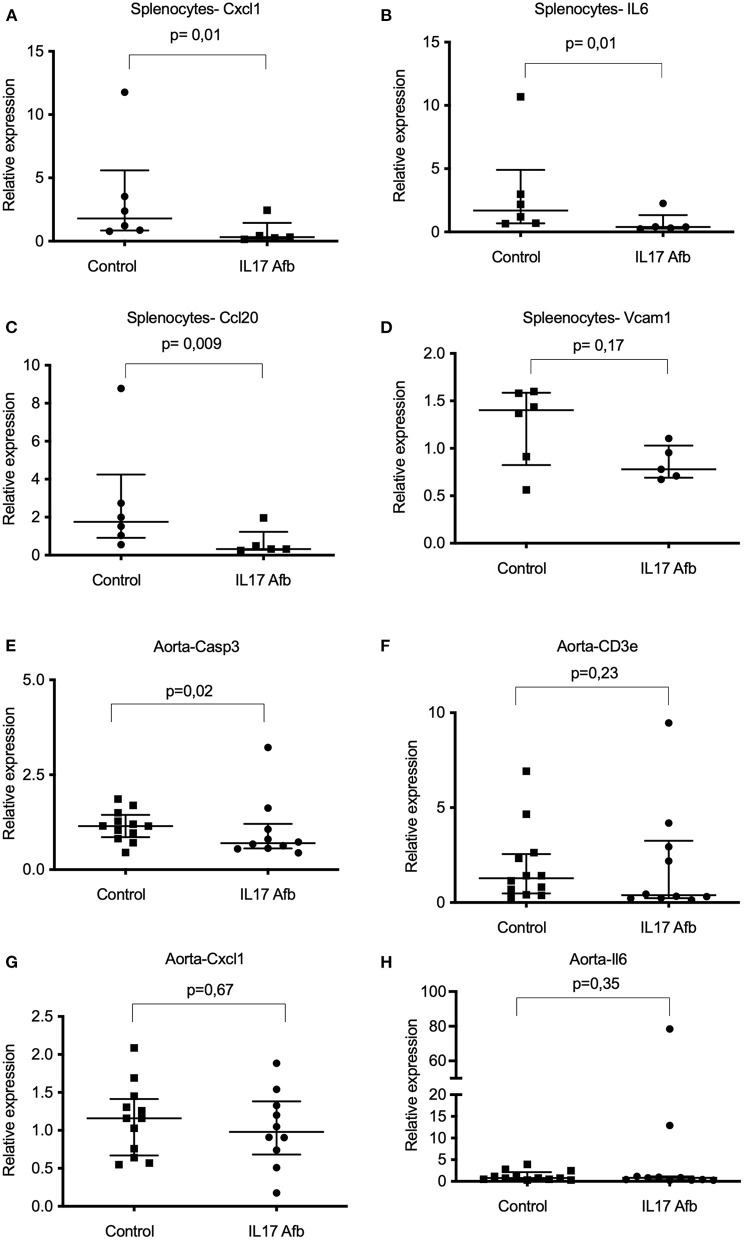
Effects of Affibody molecule against IL17A on gene expression in splenocytes and thoracic aorta from ApoE^−/−^ mice. mRNA levels were assessed by quantitative real-time reverse transcription polymerase chain reaction (qPCR) analysis. Analysis of *Cxcl1, Il6, Ccl20*, and *Vcam1* genes in splenocytes from sham (*n* = 6) and αIL17A Affibody molecule treated mice (*n* = 5) **(A-D)**. Analysis of *Casp3, Cd3e Cxcl1*, and *Il6* transcripts in the thoracic aorta from sham (*n* = 12) and αIL17A Affibody molecules treated mice (*n* = 10-11) **(E-H)**. Data are presented as Median with IQR. NS, Not significant.

In the spleen, mRNA levels of *Cxcl1* and *Il6*, were significantly lower in splenocytes from mice treated with αIL17A Affibody molecule compared with sham (Figures 4A,B). Further, mRNA levels of *Ccl20* were also significantly lower in splenocytes from mice treated with αIL17A Affibody molecule compared to sham (Figure 4C). No significant differences were observed in mRNA levels of *Vcam1* (Figure 4D), *Tnf* , *Il1-*β, *Ifn-*γ, *Tgf*β*1, Ccl2, Ccl5, Il-4*, and *Casp3* in splenocytes from mice treated with αIL17A Affibody molecules compared with sham (data not shown).

We observed significantly lower levels of *Casp3* mRNA in the thoracic aorta of αIL17A Affibody molecule treated mice compared with sham (Figure 4E). Reduced median level of *Cd3* mRNA was also observed in the aorta of mice treated with αIL17A (Figure 4F), but the difference did not reach statistical significance. In contrast to the observations in splenocytes, mRNA levels of *Cxcl1* and *Il6* were not significantly different in the aorta of αIL17A Affibody molecule treated mice compared to sham (Figures 4G,H). Further, mRNA levels of *Il1-*β*, Ifn-*γ*, Tgf*β*1, Ccl2, Ccl5, Vcam1*, and *Col1a1* were not significantly different in the aorta of αIL17A treated mice compared to sham (data not shown).

## Discussion

In the present study, we investigate the effect of αIL17A Affibody molecule and found that αIL17A Affibody molecule reduced IL6 and CXCL1 release by human smooth muscle cells and murine NIH/3T3 fibroblasts. *In vivo-*administration of αIL17A Affibody molecule in atherosclerosis-prone ApoE^−/−^ mice attenuated plasma levels of inflammatory markers and downregulated Th17 associated genes in splenocytes. There was no significant effect on atherosclerosis burden. The reduced levels of CXCL1 by exposure to or treatment with IL17A blockade observed here are consistent with the findings by Smith et al. who reported that blockade of IL17A reduced circulating CXCL1 levels in ApoE^−/−^ mice (6). Together with the observations of reduced HGF, PDGBF, MAP2K6, and QDPR, these results suggest that the novel Affibody molecule effectively blocks IL17A and attenuates circulating inflammatory markers and other atherosclerosis-associated proteins.

In this study, mice treated with the Affibody molecule against IL17A showed normal growth and development. Of note, we found elevated plasma protein levels of glucagon in mice treated with Affibody molecule against IL17A. Glucagon counteracts insulin action by stimulating hepatic glucose production and thus increasing blood glucose levels (21). However, the effect was not prominent here since we did not observe any significant changes in plasma glucose levels between groups. In connection with this, it has been reported that IL17A negatively regulates adipogenesis and glucose metabolism in mice and delays obesity development (22). No significant difference in body weight was observed between the groups in this present study. In line with these observations, plasma cholesterol, HDL, LDL, and triglyceride levels were not significantly different between groups, which is in accordance with previous studies of IL17A blocking in ApoE^−/−^ mice (10, 23). Together, the net effect on feeding and metabolism in this study was neutral as we found no evidence here that the Affibody treatment impacted growth or systemic metabolism.

Several experimental studies of IL17A in atherosclerosis show conflicting results (6, 9, 10, 23–27). The differences in results are likely, at least in part, explained by differences in study design, use of neutralizing antibodies, genetic background of mice, and type of diet. Here, we determined the IL17-blocking treatment regimen based on an *in silico* simulation of *in vivo* Affibody concentrations. This was performed in order to achieve a sufficient trough concentration between injections to maintain IL17 blocking.

The mice were housed in a facility with extensive experience of experimental atherosclerosis research and were fed a chow diet. We observed no significant differences between the experimental groups of ApoE^−/−^ mice in atherosclerotic lesion development. A possible explanation for the lack of significant effects on atherosclerosis burden by the Affibody administered here is the time course used for treatment, the dose and frequency chosen of Affibody administration, and the status of disease development at study initiation. Of note, this study addresses early stage atherosclerosis. In light of the conflicting results of previous studies and the lack of significant effect on atherosclerosis development by the Affibody directed against IL-17 activity investigated here, future studies are needed to detail the effect of IL-17 blocking at all stages of atherosclerosis.

IL17A is known to promote the release of IL6 (7, 14, 15), but there was no significant effect on plasma IL6 levels in ApoE^−/−^ mice treated with αIL17A Affibody molecule in this study. One possible explanation for this is the time point of observation here. As tissues and blood were collected 1 week after the last injection of the Affibody molecule, we speculate that there was no sustained blockade of IL17A resulting from the treatment at that time. Our observations agree with those reported by Cheng et al., who in their experiments found no significant changes in serum IL6 levels 1 week after the last injection with IL17A blocking antibody in ApoE^−/−^ mice (23).

An important limitation of this study is that we, for technical reasons, were only able to study aortic root atherosclerosis in a small number of samples. We observed in this limited sample that the size of the atherosclerotic plaques was lower in Affibody-treated compared with sham-treated mice. We report this observation for full transparency, but it should be underscored that the sample size is not suitable for firm conclusions. It is tempting to speculate that effects of this Affibody on atherosclerosis in ApoE-/- mice may be discrepant between segments in the arterial tree, but the sample size here is too limited and further studies are needed.

In conclusion, our study reports that a novel Affibody molecule targeting IL17A reduced IL6 and CXCL1 release by human SMCs and NIH/3T3 cells. Injecting Affibody molecules against IL17A in ApoE^−/−^ mice attenuated levels of pro-inflammatory mediators in plasma and downregulated Th17-associated transcripts in splenocytes. Together, these findings suggest that the novel Affibody molecule blocks IL17A. In light of the available data on IL17A-blocking in the pathogenesis of atherosclerosis and cardiovascular disease, it will be important to confirm the present observations in independent future studies and, depending on the findings in subsequent studies, consider whether it would be prudent to explore further the clinically used human-specific only affibody molecule ABY-035 as a potential therapeutic agent in blocking IL17A in subjects with atherosclerosis.

## Data Availability Statement

The original contributions presented in the study are included in the article/Supplementary Material, further inquiries can be directed to the corresponding author/s.

## Ethics Statement

The animal study was reviewed and approved by Regional Ethical Committee on Animal Experiments Stockholm.

## Author Contributions

AK carried out the experiments, data analysis, and drafted the manuscript. AK and AS participated in the creation of study design, coordination, data analysis, and finalization of manuscript. MZ and GP participated in performing experiments, data analysis, and writing—review and editing. AG and DK participated in formal analysis, investigation, and writing—review and editing. RB participated in performing experiments and data analysis. OA, SH, HJ, and LS participated in performing experiments. AC participated in performing experiments and writing—review and editing. LG and FF contributed to scientific rational, molecular design and generation of the Affibody molecule, and writing—review and editing. LL participated in formal analysis and writing—review and editing. PO participated in writing—review and editing. All authors read and approved the final manuscript.

## Funding

This work was supported by the Knowledge Foundation (KKHÖG 20150245). AK was supported by grant from the Knowledge Foundation (KKHÖG 20150245). DK and RB were supported by grants from the Swedish Heart-Lung Foundation and the Novo Nordisk Foundation (NNF15CC0018346). PO was supported by grants from the Swedish Heart-Lung Foundation, the Swedish Research Council, the Knut and Alice Wallenberg Foundation, ALF/The Stockholm Region, and MedTechLabs.

## Conflict of Interest

LG and FF are employees of Affibody AB. PO is a shareholder of Emune AB. The remaining authors declare that the research was conducted in the absence of any commercial or financial relationships that could be construed as a potential conflict of interest.

## Publisher's Note

All claims expressed in this article are solely those of the authors and do not necessarily represent those of their affiliated organizations, or those of the publisher, the editors and the reviewers. Any product that may be evaluated in this article, or claim that may be made by its manufacturer, is not guaranteed or endorsed by the publisher.

## References

[1] Tabas I Lichtman AH. Monocyte-macrophages and T Cells in atherosclerosis. Immunity. (2017) 47:621-34. 10.1016/j.immuni.2017.09.008 29045897 PMC5747297

[2] Witztum JL Lichtman AH. The influence of innate and adaptive immune responses on atherosclerosis. Annu Rev Pathol. (2014) 9:73-102. 10.1146/annurev-pathol-020712-163936 23937439 PMC3988528

[3] Bettelli E Korn T Oukka M Kuchroo VK. Induction and effector functions of T(H)17 cells. Nature. (2008) 453:1051-7. 10.1038/nature07036 18563156 PMC6280661

[4] Roark CL Simonian PL Fontenot AP Born WK O'Brien RL. Gamma delta T cells: an important source of IL-17. Curr Opin Immunol. (2008) 20:353-7. 10.1016/j.coi.2008.03.006 18439808 PMC2601685

[5] Li L Huang L Vergis AL Ye H Bajwa A Narayan V. IL-17 produced by neutrophils regulates IFN-gamma-mediated neutrophil migration in mouse kidney ischemia-reperfusion injury. J Clin Invest. (2010) 120:331-42. 10.1172/JCI38702 20038794 PMC2798679

[6] Smith E Prasad KM Butcher M Dobrian A Kolls JK Ley K. Blockade of interleukin-17A results in reduced atherosclerosis in apolipoprotein E-deficient mice. Circulation. (2010) 121:1746-55. 10.1161/CIRCULATIONAHA.109.924886 20368519 PMC2929562

[7] Eid RE Rao DA Zhou J Lo SF Ranjbaran H Gallo A. Interleukin-17 and interferon-gamma are produced concomitantly by human coronary artery-infiltrating T cells and act synergistically on vascular smooth muscle cells. Circulation. (2009) 119:1424-32. 10.1161/CIRCULATIONAHA.108.827618 19255340 PMC2898514

[8] Gao Q Jiang Y Ma T Zhu F Gao F Zhang P. A critical function of Th17 proinflammatory cells in the development of atherosclerotic plaque in mice. J Immunol. (2010) 185:5820-7. 10.4049/jimmunol.1000116 20952673 PMC12230985

[9] Madhur MS Funt SA Li L Vinh A Chen W Lob HE. Role of interleukin 17 in inflammation, atherosclerosis, and vascular function in apolipoprotein e-deficient mice. Arterioscler Thromb Vasc Biol. (2011) 31:1565-72. 10.1161/ATVBAHA.111.227629 21474820 PMC3117048

[10] Erbel C Chen L Bea F Wangler S Celik S Lasitschka F. Inhibition of IL-17A attenuates atherosclerotic lesion development in apoE-deficient mice. J Immunol. (2009) 183:8167-75. 10.4049/jimmunol.0901126 20007582

[11] Stahl S Graslund T Eriksson Karlstrom A Frejd FY Nygren PA Lofblom J. Affibody molecules in biotechnological and medical applications. Trends Biotechnol. (2017) 35:691-712. 10.1016/j.tibtech.2017.04.007 28514998

[12] Yu F Gudmundsdotter L Akal A Gunneriusson E Frejd F Nygren PA. An affibody-adalimumab hybrid blocks combined IL-6 and TNF-triggered serum amyloid A secretion *in vivo*. MAbs. (2014) 6:1598-607. 10.4161/mabs.36089 25484067 PMC4622551

[13] Centa M Ketelhuth DFJ Malin S Gistera A. Quantification of atherosclerosis in mice. J Vis Exp. (2019) 148. 10.3791/59828 31259915

[14] Fossiez F Djossou O Chomarat P Flores-Romo L Ait-Yahia S Maat C. T cell interleukin-17 induces stromal cells to produce proinflammatory and hematopoietic cytokines. J Exp Med. (1996) 183:2593-603. 10.1084/jem.183.6.2593 8676080 PMC2192621

[15] Taleb S Tedgui A Mallat Z. IL-17 and Th17 cells in atherosclerosis: subtle and contextual roles. Arterioscler Thromb Vasc Biol. (2015) 35:258-64. 10.1161/ATVBAHA.114.303567 25234818

[16] Bell EJ Decker PA Tsai MY Pankow JS Hanson NQ Wassel CL. Hepatocyte growth factor is associated with progression of atherosclerosis: the multi-ethnic study of atherosclerosis (MESA). Atherosclerosis. (2018) 272:162-7. 10.1016/j.atherosclerosis.2018.03.040 29609131 PMC5908230

[17] Gallo S Sala V Gatti S Crepaldi T. Cellular and molecular mechanisms of HGF/Met in the cardiovascular system. Clin Sci. (2015) 129:1173-93. 10.1042/CS20150502 26561593

[18] Munzel T Daiber A. Role of endothelial and macrophage tetrahydrobiopterin in development and progression of atherosclerosis: BH4 puzzle solved? Cardiovasc Res. (2018) 114:1310-2. 10.1093/cvr/cvy118 29878064

[19] Reustle A Torzewski M. Role of p38 MAPK in atherosclerosis and aortic valve sclerosis. Int J Mol Sci. (2018) 19:3761. 10.3390/ijms19123761 30486366 PMC6321637

[20] Zhang Y Zhang W Wang KQ Li T Song SH Yuan B. Expression of platelet-derived growth factor in the vascular walls of patients with lower extremity arterial occlusive disease. Exp Ther Med. (2015) 9:1223-8. 10.3892/etm.2015.2275 25780413 PMC4353744

[21] Wewer Albrechtsen NJ Kuhre RE Pedersen J Knop FK Holst JJ. The biology of glucagon and the consequences of hyperglucagonemia. Biomark Med. (2016) 10:1141-51. 10.2217/bmm-2016-0090 27611762

[22] Zuniga LA Shen WJ Joyce-Shaikh B Pyatnova EA Richards AG Thom C. IL-17 regulates adipogenesis, glucose homeostasis, and obesity. J Immunol. (2010) 185:6947-59. 10.4049/jimmunol.1001269 21037091 PMC3001125

[23] Cheng X Taleb S Wang J Tang TT Chen J Gao XL. Inhibition of IL-17A in atherosclerosis. Atherosclerosis. (2011) 215:471-4. 10.1016/j.atherosclerosis.2010.12.034 21300351

[24] van Es T van Puijvelde GH Ramos OH Segers FM Joosten LA van den Berg WB. Attenuated atherosclerosis upon IL-17R signaling disruption in LDLr deficient mice. Biochem Biophys Res Commun. (2009) 388:261-5. 10.1016/j.bbrc.2009.07.152 19660432

[25] Gistera A Robertson AK Andersson J Ketelhuth DF Ovchinnikova O Nilsson SK. Transforming growth factor-beta signaling in T cells promotes stabilization of atherosclerotic plaques through an interleukin-17-dependent pathway. Sci Transl Med. (2013) 5:196ra00. 10.1126/scitranslmed.3006133 23903754

[26] Butcher MJ Gjurich BN Phillips T Galkina EV. The IL-17A/IL-17RA axis plays a proatherogenic role *via* the regulation of aortic myeloid cell recruitment. Circ Res. (2012) 110:675-87. 10.1161/CIRCRESAHA.111.261784 22302786 PMC3337709

[27] Danzaki K Matsui Y Ikesue M Ohta D Ito K Kanayama M. Interleukin-17A deficiency accelerates unstable atherosclerotic plaque formation in apolipoprotein E-deficient mice. Arterioscler Thromb Vasc Biol. (2012) 32:273-80. 10.1161/ATVBAHA.111.229997 22116098

