# Supplementary material for: Inhibition of IL17A Using an Affibody Molecule Attenuates Inflammation in ApoE-Deficient Mice
Source: Front Cardiovasc Med. 2022 Feb 24;9:831039. doi: 10.3389/fcvm.2022.831039 (PMC8907570; doi:10.3389/fcvm.2022.831039)
Supplement: Supplementary file 1 [file Data_Sheet_1.docx]

**Supplementary Figure 1**

***Supplementary Figure 1.*** *Affibody molecule against IL17A reduces plasma levels of inflammatory and/or atherosclerosis-associated proteins in ApoE^-/-^ mice.*

Plasma protein levels were analysed by olink proteomics in ApoE^-/-^ mice (n = 11-12). Linear normalized protein expression (NPX) values are shown. Data are presented as Median with IQR.

**Supplementary Figure 2**

***Supplementary Figure 2.*** *Plasma levels of inflammatory markers in ApoE^-/-^ mice.*

Plasma protein levels were analysed by olink proteomics in ApoE^-/-^ mice (n = 11-12). Linear normalized protein expression (NPX) values are shown. Data are presented as Median with IQR.

**Supplementary Figure 3**

***Supplementary Figure 3.*** *Plasma protein levels of inflammatory markers in ApoE^-/-^mice.*

Plasma protein levels were analysed olink proteomics in ApoE^-/-^ mice (n = 11-12). Linear normalized protein expression (NPX) values are shown. Data are presented as Median with IQR.

***Supplementary Table 1.***

Primer sequences, forward (Fwr) and reverse (Rev) primer are shown.

| Name | Sequences |
| --- | --- |
| *Cd3e Fwr Seq* | 5'-GCTCCAGGATTTCTCGGAAGTC-3' |
| *Cd3e Rev Seq* | 5'-ATGGCTACTGCTGTCAGGTCCA-3' |
| *Ccl2 Fwr Seq* | 5'-GCTACAAGAGGATCACCAGCAG-3' |
| *Ccl2 Rev Seq* | 5'-GTCTGGACCCATTCCTTCTTGG-3' |
| *Ccl5 Fwr Seq* | 5'-CCTGCTGCTTTGCCTACCTCTC-3' |
| *Ccl5 Rev Seq* | 5'-ACACACTTGGCGGTTCCTTCGA-3' |
| *Ccl20 Fwr Seq* | 5'-GTGGGTTTCACAAGACAGATGGC-3' |
| *Ccl20 Rev Seq* | 5'-CCAGTTCTGCTTTGGATCAGCG-3' |
| *Cxcl1 Fwr Seq* | 5'-TCCAGAGCTTGAAGGTGTTGCC-3' |
| *Cxcl1 Rev Seq* | 5'-AACCAAGGGAGCTTCAGGGTCA-3' |
| *Il1β Fwr Seq* | 5'-TGGACCTTCCAGGATGAGGACA-3' |
| *Il1β Rev Seq* | 5'-GTTCATCTCGGAGCCTGTAGTG-3' |
| *Il4 Fwr Seq* | 5'-CATCGGCATTTTGAACGAG-3' |
| *Il4 Rev Seq* | 5'-CGAGCTCACTCTCTGTGGTG-3' |
| *Il6 Fwr Seq* | 5'-TACCACTTCACAAGTCGGAGGC-3' |
| *Il6 Rev Seq* | 5'-CTGCAAGTGCATCATCGTTGTTC-3' |
| *IFNγ Fwr Seq* | 5'-TCTGGAGGAACTGGCAAAAG-3' |
| *IFNγ Rev Seq* | 5'-TTCAAGACTTCAAAGAGTCTGAGG-3' |
| *Tnf Fwr Seq* | 5'-ACCCTCACACTCAGATCATCTTC-3' |
| *Tnf Rev Seq* | 5'-TGGTGGTTTGCTACGACGT-3' |
| *Tgfβ1 Fwr Seq* | 5'-GGACTCTCCACCTGCAAGAC -3' |
| *Tgfβ1 Rev Seq* | 5'-GACTGGCGAGCCTTAGTTTG -3' |
| *Vcam1 Fwr Seq* | 5'-AGTTGGGGATTCGGTTGTTCT-3' |
| *Vcam1 Rev Seq* | 5'-CCCCTCATTCCTTACCACCC-3' |
| *Caspase3 Fwr Seq* | 5'-GGAGTCTGACTGGAAAGCCGAA-3' |
| *Caspase3 Rev Seq* | 5'-CTTCTGGCAAGCCATCTCCTCA-3' |
| *Col1a1 Fwr Seq* | 5'-CCTCAGGGTATTGCTGGACAAC-3' |
| *Col1a1 Rev Seq* | 5'-CAGAAGGACCTTGTTTGCCAGG-3' |
| *Actb Fwr Seq* | 5'-CATTGCTGACAGGATGCAGAAGG-3' |
| *Actb Rev Seq* | 5'-TGCTGGAAGGTGGACAGTGAGG-3' |
|  |  |
